# The impact of sequential therapy from short-term teriparatide to denosumab compared with denosumab alone in patients with osteoporotic hip fracture: a 1-year follow-up study

**DOI:** 10.1186/s12891-020-03771-8

**Published:** 2020-11-14

**Authors:** Chan Ho Park, Jun-Il Yoo, Chang Hyun Choi, You-Sung Suh

**Affiliations:** 1 Department of Orthopedic Surgery, Yeungnam University Medical Center, 170, Hyeonchung-ro, Nam-gu, Daegu, 42415 South Korea; 2 Department of Orthopaedic Surgery, Gyeongsang National University Hospital, 79, Gangnam-ro, Jinju, Gyeongsangnam-do 52727 South Korea; 3 Department of Orthopaedic Surgery, Soonchunhyang University Seoul Hospital, 59, Daesagwan-ro, Yongsan-gu, Seoul, 04401 South Korea

**Keywords:** Teriparatide, Denosumab, Osteoporosis, Hip fracture, Sequential therapy

## Abstract

**Background:**

Sequential therapy from bone-forming medication to resorptive agents is reportedly effective for patients with severe osteoporosis. The objective of this study is to determine the impact of implementing short-term teriparatide (TPTD) intervention before denosumab (DMab) therapy compared with DMab therapy alone for 1 year after hip fracture.

**Methods:**

We retrospectively reviewed the medical records and radiographs of patients who were treated due to osteoporotic hip fracture. TPTD was administered to 22 patients for an average of 12.1 weeks after which the intervention was switched to DMab therapy for 12 months (group 1). DMab alone was administered to 16 patients for 12 months (group 2). Bone mineral density (BMD) was evaluated before and after treatment at the 1-year follow-up. The improvement of BMD in hip and spine was compared with the levels of bone turnover marker.

**Results:**

The difference in femoral neck BMD was 0.005 ± 0.04 in group 1 and − 0.014 ± 0.10 in group 2 (*p* = 0.442). The difference of spine BMD was 0.043 ± 0.05 in group 1 and 0.052 ± 0.06 in group 2 (*p* = 0.640). BMD of the spine improved significantly in groups 1 and 2 (*p* < 0.001, *p* = 0.002). There was no statistical difference in C-terminal telopeptide and osteocalcin level.

**Conclusion:**

Short-term TPTD administration followed by DMab alone was effective only in improving spine BMD. Short-term treatment with TPTD caused mild improvement in femur neck BMD compared with DMab alone. However, further research with a longer duration of TPTD treatment is warranted, as our findings lack statistical significance.

## Background

Second hip fracture is defined as the occurrence of fracture in contralateral hip subsequent to hip fracture on one side [1–3]. Although the incidence of second hip fracture was reported as 1.7–14.8% in previous studies, the mortality rate in patients with second hip fracture is very high compared to that in patients sustaining primary hip fracture alone [4]. Therefore, the treatment for osteoporosis is critical to help patients with primary hip fracture to prevent recurrence.

Anti-resorptive agents have been the mainstay of osteoporosis treatment. Antiresorptive agents are categorized into bisphosphonate (BP) and denosumab (DMab) largely depending on the mechanism of inhibition of osteoclastic bone resorption [5, 6]. Despite long-term treatment with BPs, bone mineral density (BMD) reaches plateaus, thereby increasing the risk of atypical femur fracture [7]. However, DMab was approved by United States Food and Drug Administration in 2010, as it helps improve BMD and prevent fracture [8]. In contrast to BP, long-term DMab therapy is associated with continuous improvement in BMD [9].

Teriparatide (TPTD) is a bone-forming agent [10] that increases the formation of new bone tissue and partly resolves the structural defects in the osteoporotic bone [11]. Moreover, the effect of bone union after fracture is uncertain although a few clinical trials showed enhanced bone union [12].

Combination therapy of anti-resorptive and bone-forming agents wherein one agent is independently administered for a specific period of a time after which the other agent is administered is a very effective option for patients diagnosed with severe osteoporotic hip fracture as it improves BMD and enhances bone healing. The Denosumab and Teriparatide Transitions in Postmenopausal Osteoporosis (The DATA-Switch Study) showed that combination therapy using DMab following DMab and TPTD for 2 years, and sequential therapy using DMab following TPTD for 2 years resulted in excellent BMD improvement [13]. However, adherence with combination and sequential therapies for 2 years is exceedingly difficult because of economic burden and daily injection complaints of TPTD. Nevertheless, aggressive treatment options for osteoporosis such as combination and sequential therapies are needed for patients with hip fracture to rapidly improve BMD and prevent second hip fracture.

Therefore, the purpose of this study was to determine the impact of sequential therapy using short-term teriparatide (TPTD) intervention before denosumab (DMab) therapy compared with DMab therapy alone for 1 year after hip fracture.

## Methods

The present study protocol was reviewed and approved by the Institutional Review Board of Yeungnam University Hospital (approval No. YUMC 2019–10-025). The need to obtain informed consent was waived because of the retrospective nature of the study. From September 2016 and April 2018, when DMab therapy began to be available in Korea, we reviewed the medical records and radiographs of patients treated by DMab among patients who underwent operation for hip fracture at 2 tertiary referral hospitals. The inclusion criteria were (1) female patients aged over 55 years with fragility hip fracture due to low energy trauma like simple fall and (2) followed-up for a minimum of 1-year with dual-energy X-ray absorptiometry (DEXA) scan after hip fracture surgery. We excluded patients with secondary hip fracture.

**Fig. 1:**
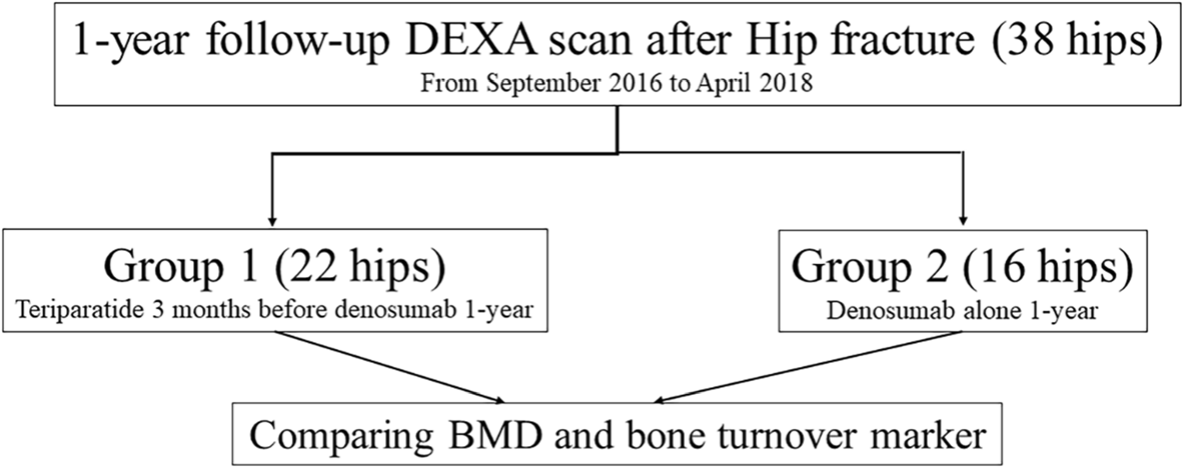
Flow chart of this study

During the study period, 38 patients treated with DMab for 1 year underwent DEXA scan. We divided them into two groups to determine the effect of short-term sequential therapy. TPTD was administered to 22 patients for an average of 12.1 weeks (range 8–20 weeks) before switching to DMab for 12 months (group 1). DMab alone was administered to 16 patients for 12 months (group 2). (Fig. 1).

Serum C-terminal telopeptide (CTX) and osteocalcin were evaluated in all patients before treatment for osteoporosis and after 1 year of treatment. All patients were supplemented with calcium and vitamin D during the study period.

The primary outcome measure was the intergroup difference in BMD posttreatment and bone turnover marker. Secondary outcome was BMD improvement in each group. Independent and paired t-tests were used for univariate analysis of continuous variables. Differences were considered significant if *p* values were < 0.05. All analyses were performed using SPSS version 20.0 for Windows (SPSS Inc., Chicago, IL).

## Results

Group 1 consisted of 22 women, and the mean age of patients at the time of surgery was 75.9 ± 6.06 years (range 60–88 years). Group 2 comprised 16 women, whose mean age at the time of surgery was 75.4 ± 7.94 years (range 64–86 years). There was no statistically significant difference in baseline characteristics between the two groups. However, the level of vitamin D was significantly lower in group 2 than in group 1 (*p* = 0.025) (Table 1).

**Table 1:** Demographic findings of this study

Variables	Group 1 (TPTD+DMAb)	Group 2 (DMAb)	*p*-value
Number	22	16	
Age (years)			
Mean ± SD	75.9 ± 6.06	75.4 ± 7.94	0.831
Diagnosis (N)			
ITN/FN/STN	17/4/1	7/9/0	0.06
PTH (ng/ml)			
Mean ± SD	43.44 ± 22.05	42.94 ± 20.38	0.951
Vit D (ng/ml)			
Mean ± SD	19.37 ± 15.20	8.23 ± 4.04	0.025
Initial Hip BMD (g/cm^3^)			
Mean ± SD	0.650 ± 0.11	0.626 ± 0.11	0.506
Initial Spine BMD (g/cm^3^)			
Mean ± SD	0.735 ± 0.14	0.702 ± 0.11	0.420

*ITN* intertrochanter fracture, *FN* femur neck fracture, *STN* subtrochanter fracture, *PTH* parathyroid hormone, *BMD* bone mineral density

The difference in value of hip BMD calculated before and after osteoporosis treatment was − 0.01 ± 0.03 in group 1 and 0.007 ± 0.04 in group 2 (*p* = 0.147). The difference in femoral neck BMD was 0.005 ± 0.04 in group 1 and − 0.014 ± 0.10 in group 2 (*p* = 0.442). The difference in spine BMD was 0.043 ± 0.05 in group 1 and 0.0515 ± 0.06 in group 2 (*p* = 0.640) (Table 2).

**Table 2:** The difference of bone mineral density and bone turnover marker during study period

	Difference between before and after treatment (mean ± SD)	*p*-value
Hip BMD (g/cm^2^)		
group 1	−0.01 ± 0.03	0.147
group 2	0.007 ± 0.04	
Femoral neck BMD (g/cm^2^)		
group 1	0.005 ± 0.04	0.442
group 2	−0.014 ± 0.10	
Spine BMD (g/cm^2^)		
group 1	0.043 ± 0.05	0.640
group 2	0.052 ± 0.06	
Serum osteocalcin (ng/mL)		
group 1	0.39 ± 3.25	0.764
group 2	−0.33 ± 1.85	
Serum CTX (ng/mL)		
group 1	−0.30 ± 0.55	0.252
group 2	−0.09 ± 0.28	

*BMD* bone mineral density; *CTX* C-terminal telopetide

**Fig. 2:**
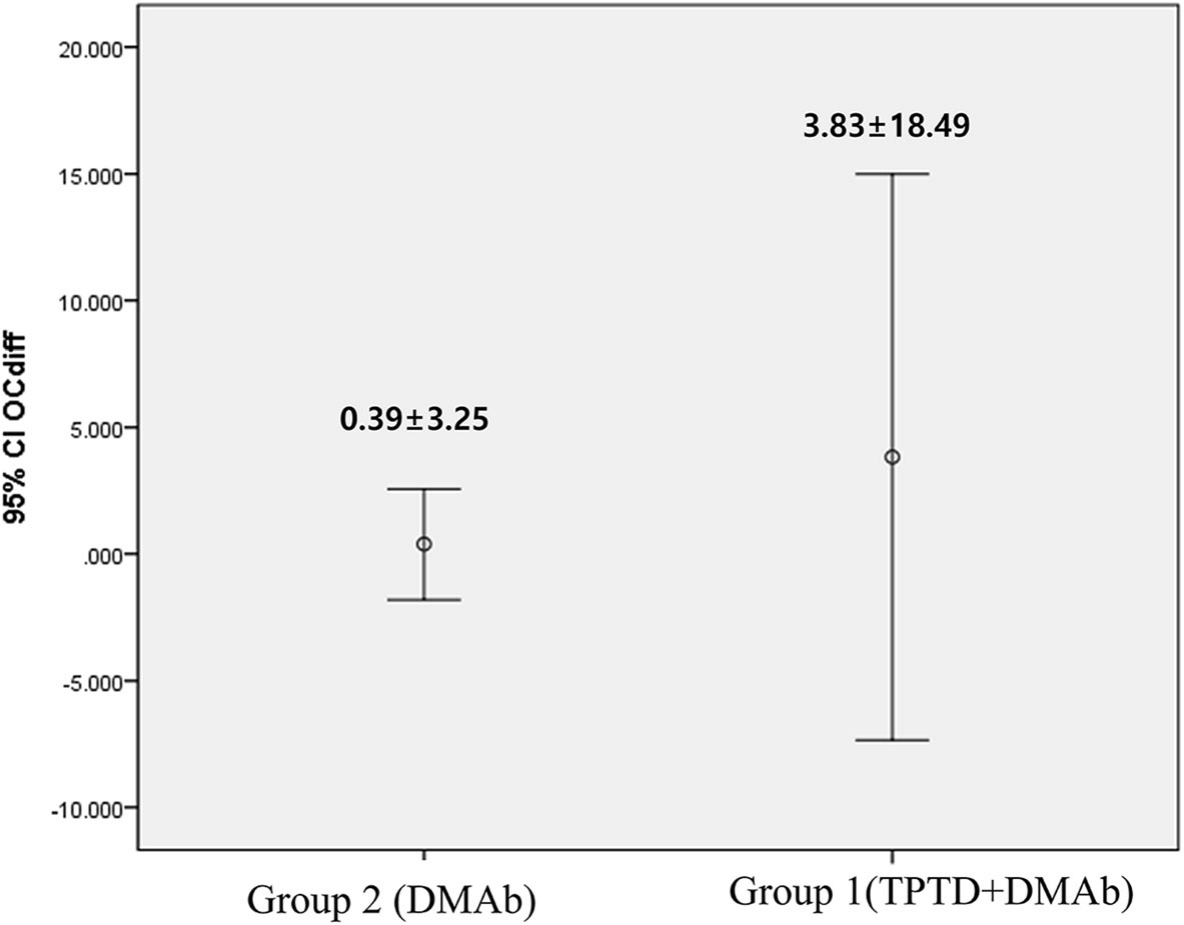
The change in serum osteocalcin following treatment

**Fig. 3:**
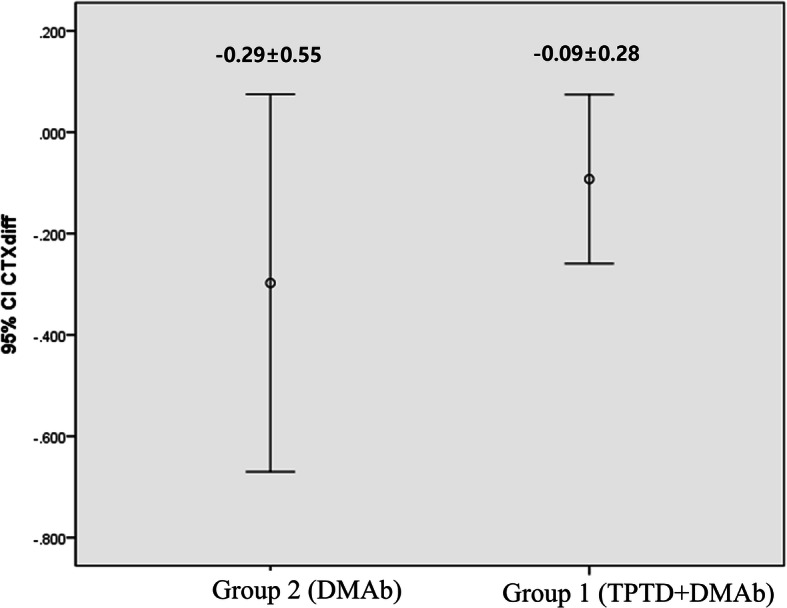
The change in serum C-terminal telopeptide for 1-year follow-up

During the one-year follow-up period, there were no statistically significant differences in serum CTX and osteocalcin levels in both groups (Figs. 2, 3).

The hip and femoral neck BMD improved mildly in both groups. However, the improvement was not statistically significant. However, the BMD of the spine improved significantly in groups 1 and 2 (*p* < 0.001, *p* = 0.02) (Table 3).

**Table 3:** The improvement of bone mineral density for 1 year follow up

		Immediate Post-operation (g/cm^2^)	1-year follow-up (g/cm^2^)	*p*-value
Group 1 (TPTD+DMAb)	Femoral neck	0.540 ± 0.12	0.546 ± 0.11	0.584
	Total hip	0.654 ± 0.09	0.644 ± 0.11	0.152
	Spine	0.735 ± 0.14	0.778 ± 0.13	< 0.001
Group 2 (DMAb)	Femoral neck	0.481 ± 0.11	0.467 ± 0.09	0.566
	Total hip	0.626 ± 0.11	0.633 ± 0.11	0.454
	Spine	0.702 ± 0.11	0.753 ± 0.78	0.002

*TPTD* teriparatide, *DMAb* denosumab

## Discussion

Short-term sequential therapy, DMab therapy after a short-term TPTD therapy, did not significantly change hip BMD and bone turnover marker in patients with severe osteoporotic hip fracture. However, DMab therapy regardless of short-term use of TPTD significantly improved spine BMD in both groups. This might be due to the difference in bone composition. Generally, BMD of the spine, which consists mostly cancellous bone, improved after osteoporotic treatment. TPTD is well known for improving cancellous bone density and reducing cortical porosity [14, 15]. Therefore, the short term TPTD therapy was able to affect cancellous bone, whereas it was too short to reduce cortical porosity. In order to ensure fracture healing in patients with hip fracture, the process of bone formation should be adequately established in the early stages of fracture healing. Because the femur necks are composed mainly of cortical bones, the use of appropriate bone-forming agents is important for fracture healing. Therefore, previous studies reported sequential or combination treatment involving TPTD and denosumab therapies [13, 16, 17].

In the DATA-Switch study, TPTD was used for 2 years, with satisfactory results reported for the treatment of high-fracture risk groups following a switch to denosumab [13].

In the real world, however, compliance with TPTD therapy is difficult due to exorbitant cost [18, 19]. Although the results of this study were not statistically significant, we found that denosumab conversion after 3 months of TPTD use may be effective in patients with a high risk of fracture.

Almirol EA et al. [20] performed the randomized placebo-control study to evaluate the short-term effect (8 weeks) of TPTD in patients with lower-extremity stress fracture. Short-term TPTD treatment showed anabolic effects suggesting that TPTD may accelerate fracture healing in premenopausal women with lower-extremity stress fractures.

Kang et al. [21] performed a prospective comparative study to determine whether 3 months of TPTD therapy may be effective for preventing of fracture progression in patients with osteoporotic vertebral compression fractures (VCF) at the thoracolumbar spine. However, they found that 3-month treatment with TPTD did not prevent the progression of fractured vertebral body collapse or kyphotic changes in patients with osteoporosis. Similar with this study, sequential therapy was used, short-term TPTD followed by switch to denosumab for 1 year in our study. Spine BMD improved but the hip BMD did not show a statistically significant difference during the 1-year follow-up. In addition, both groups showed successful bone union at 1-year follow- up.

There are several limitations to this study. First, this was a retrospective study assessing a small sample of patients. Therefore, based on the results of this study, a well-planned RCT study is needed. Second, bone turnover markers were not measured between 3 and 6 months, and the level of validation for serum markers may differ between two hospitals. Further studies are needed to evaluate the BTM levels simultaneously at a central lab. In addition, a number of studies should be assessed by adding a reference interval for Koreans, and consensus on the timing of BTM measurement after fracture. Third, there was no objective assessment of the degree of bone union. In order to accurately assess the degree of bone union, all patients should be evaluated with imaging tests, including CT scan. Fourth, baseline value of vitamin D was difference between two groups. However, that did not significantly affect the improvement of BMD because all patients were supplemented with calcium and vitamin D equally.

## Conclusion

Our findings suggest that sequential therapy using short-term TPTD and DMab treatments can effectively improve spine BMD and mildly improve femur neck BMD. Based on differences in the composition of cancellous and cortical bones of hip and spine, DMab therapy following TPTD for more than 3 months might be effective for patients with osteoporotic hip fracture who are at an increased risk of subsequent hip fracture. Well-designed prospective, multicenter studies will be needed to determine the impact of implementing short-term teriparatide.

## Data Availability

The datasets used and/or analyzed during the current study are available from the corresponding author on reasonable request.

## Abbreviations

DMab: Denosumab
BP: Bisphosphonate
BMD: Bone mineral density
TPTD: Teriparatide
CTX: C-terminal telopeptide
